# New Insights in the Amyloid-Beta Interaction with Mitochondria

**DOI:** 10.1155/2012/324968

**Published:** 2012-03-19

**Authors:** Carlos Spuch, Saida Ortolano, Carmen Navarro

**Affiliations:** Department of Pathology and Neuropathology, Hospital of Meixoeiro, University Hospital of Vigo, Meixoeiro s/n, 36215 Vigo, Spain

## Abstract

Biochemical and morphological alterations of mitochondria may play an important role in the pathogenesis of Alzheimer's disease (AD). Particularly, mitochondrial dysfunction is a hallmark of amyloid-beta-induced neuronal toxicity in Alzheimer's disease. The recent emphasis on the intracellular biology of amyloid-beta and its precursor protein (APP) has led researchers to consider the possibility that mitochondria-associated and mitochondrial amyloid-beta may directly cause neurotoxicity. Both proteins are known to localize to mitochondrial membranes, block the transport of nuclear-encoded mitochondrial proteins to mitochondria, interact with mitochondrial proteins, disrupt the electron transport chain, increase reactive oxygen species production, cause mitochondrial damage, and prevent neurons from functioning normally. In this paper, we will outline current knowledge of the intracellular localization of amyloid-beta. Moreover, we summarize evidence from AD postmortem brain as well as animal AD models showing that amyloid-beta triggers mitochondrial dysfunction through a number of pathways such as impairment of oxidative phosphorylation, elevation of reactive oxygen species production, alteration of mitochondrial dynamics, and interaction with mitochondrial proteins. Thus, this paper supports the Alzheimer cascade mitochondrial hypothesis such as the most important early events in this disease, and probably one of the future strategies on the therapy of this neurodegenerative disease.

## 1. Introduction

Each year, over 10 million people globally suffer from neurodegenerative diseases. This figure is expected to grow by 20% over the next decade as the aging population increases and lives longer. This disease group is the fourth biggest killer in the developed world after heart diseases, cancer, and stroke [1]. The most common neurodegenerative diseases are AD, Parkinson disease, Lewy body dementia, frontotemporal dementia, and amyotrophic lateral sclerosis [2]. The most widely recognized is AD, which is among the principal debilitating conditions of the current century. Approximately 24 million people worldwide suffer from dementia, 60% of cases being due to AD, which occurs in 1% of individuals aged 50 to 70 years old and dramatically increases to 50% of those over 70 years old [3]. Dramatically, these numbers are estimated to increase to 15 million in the next 40 years [4].

From the neuropathological point of view, AD is characterized by selective neuronal loss, marked synaptic alteration, morphological mitochondrial abnormalities, and Tau pathology. The histological hallmark lesions of AD are characterized by senile plaques and cerebrovascular deposits. The extracellular amyloid plaques mainly composed of amyloid-beta and intracellular neurofibrillary tangles built up of hyperphosphorylated tau, although the molecular mechanisms underlying the disease are still unknown and there is still no cure. Many lines of evidence suggest that oxidative stress is one of the earliest changes and plays an important role in the pathological process in AD, and more recently, energy deficiency and mitochondrial dysfunction have been recognized as a prominent, early event in AD [1–11]. Oxidative stress plays a critical role in the pathogenesis of AD and is intimately linked to aging, the best established risk factor for AD. Increased oxidative stress levels have been found in the brains of patients with AD in Sweden and early in Tg2576 APP transgenic mice [12, 13]. Recently, we described that PLA2G3 gene silencing produced a marked inhibition of the free radical-generating xanthine/xanthine oxidase- (X-XOD-) system-induced cell death, and that PLA2G3 polymorphisms are associated with AD in a Spanish case-control sample [14]. In previous studies with choroid plexus homogenates with different cases with AD in the different stages (I/0, III-IV, and V-VI), we demonstrated that in AD patients, amyloid-beta peptide also accumulates in choroid plexus [15], there is an oxidation of carboxymethyl-lysine (CML), and N-carboxyethyl-lysine (CEL) may result in impaired protein interactions, protein folding, and protein kinase activity; abnormal function of endothelial and vascular smooth muscle cells; impaired HDL-cholesterol metabolism in the choroid plexus in advanced stages of AD [16].

## 2. Mitochondria

Mitochondria are found in virtually all eukaryotic cells and function to generate cellular energy in the form of adenosine triphosphate (ATP) by oxidative phosphorylation and are thought to be derived evolutionarily from the fusion of prokaryotic and eukaryotic organisms [17]. They are also involved in regulation of cell death via apoptosis, in the control of cell division and growth, in calcium homeostasis, haem biosynthesis, and in the formation and export of iron-sulphur clusters.

**Figure 1:**
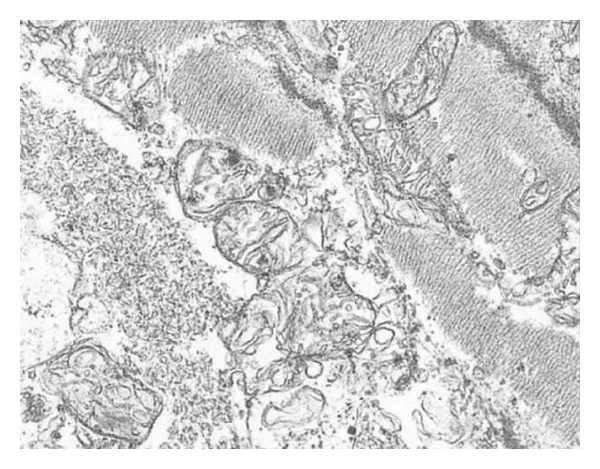
Representative electromicrographs of mitochondrial ultrastructure. Scale bar 200 nm.

Mitochondria are composed of a double lipid membrane which structures four compartments, distinct by composition and function (Figure 1). The porous outer membrane encompasses the whole organelle. It contains many proteins like import complexes and voltage-dependent anion channels responsible for the free passage of low-molecular-weight substances (up to 5000 Da) between the cytoplasm and the intermembrane space which represents a reservoir of protons establishing a proton electrochemical gradient across inner mitochondrial membrane that is needed for the production of ATP via ATPase (complex V). Intermembrane space contains proapoptotic proteins like cytochrome c, Smac/Diablo, EndoG, and Htra2/Omi. In contrast to the permeable outer membrane, the inner mitochondrial membrane, rich in cardiolipin, provides a highly efficient barrier to the flow of small molecules and ions, including protons. This membrane is invaginated into numerous cristae increasing cell surface area. It houses the respiratory enzymes of the electron transport chain, the cofactor coenzyme Q, and many mitochondrial carriers. In the matrix, different metabolic pathways take place including the tricarboxylic or Krebs cycle [18].

Mitochondria are unique amongst cellular organelles in that they have their own, circular, double-stranded DNA (mtDNA) which is inherited almost exclusively down the maternal line and codes for 37 mitochondrial genes, 13 of which translate to proteins involved in oxidative phosphorylation [18, 19]. The remaining genes encode transfer (22 genes) and ribosomal (2 genes) RNA allowing the mitochondria to generate their own proteins. Although mitochondria have the ability to produce proteins, the vast majority of proteins that function within the mitochondria, including those involved in DNA transcription, translation, and repair, are encoded by nuclear DNA and are transported into the mitochondria from the cytosol. As mtDNA is located in the mitochondria in close proximity to the electron transport chain, it is more susceptible to damage from free radicals generated during oxidative phosphorylation [20].

Mitochondria generate energy by two closely coordinated metabolic processes: Krebs cycle and the oxidative phosphorylation (OXPHOS). OXPHOS is made up of the electron transport chain assembled in four enzymes (complex I to IV) as well as the F1F0-ATP synthase (complex V). Complexes I, III, and IV are located in inner mitochondrial membrane as integral proteins, whereas complex II is attached to the inner surface of this membrane. The function of the chain is to generate cellular energy in the form of ATP. These five enzymes of the complex are connected functionally by mobile electron acceptors and donors: ubiquinone and cytochrome c. Electrons from NADH and FADH_2_ are fed into complexes I and II, respectively. Ubiquinone Q carries electrons from both complexes to complex II, and cytochrome c does it from complex III to IV reducing molecular oxygen to water. As electrons are transferred along electron transport chain, a fixed number of protons are pumped from the matrix into inner membrane space establishing a electrochemical gradient characterized with a specific electrical potential. The redox energy drives the synthesis of ATP from ADP as protons are transported back from inner membrane space into the matrix via complex V.

## 3. Amyloid-Beta in the Cytosol

Accumulation of amyloid-beta in AD brains is thought to underlie neuronal dysfunction and memory loss, being centrally implicated in AD pathogenesis. Moreover, previous to mitochondrial accumulation of amyloid-beta showed in AD patient and AD transgenic mouse brain, the toxic amyloid-beta species has to be accumulated in the cytosol of the cells. Many studies showed amyloid-beta interaction with different receptors in the cellular membrane of the vasculature, neurons, oligodendrocytes, and glial cells where it is transported from cell surface into endosomal and lysosomal compartments [21–23]. The aberrant signalling of these receptors in AD triggered an abnormal accumulation of amyloid-beta into cytosol-inducing cellular stress underlies to neuronal dysfunction and dementia.

It is described by our group and others that these receptors can be megalin, also known as low-density lipoprotein-related protein-2 (LRP2) [24], LRP-1 [22], or RAGE (receptors for advanced glycation end products) [25]. The interaction of these receptors with amyloid-beta in neurons, microglia, and vascular cells accelerates and amplifies deleterious effects on neuronal and synaptic functions. These findings are further in line with the recently proposed hypothesis of an intracellular amyloid-beta toxicity cascade which suggests that the toxic amyloid-beta species intervening in molecular and biochemical abnormalities may be intracellular soluble aggregates instead of extracellular, insoluble plaques. There are many studies proposing that megalin- and/or RAGE-dependent signalling are involved in the regulation of amyloid-beta clearance and probably may contribute to amyloid pathology and cognitive dysfunction observed in the AD patients and AD mouse model.

## 4. Amyloid-Beta and Mitochondria

Studies of postmortem brains from AD patients and transgenic mouse models of AD suggest that oxidative damage, induced by amyloid-beta, is associated with mitochondria early in AD progression. Amyloid-beta and APP protein are known to localize to mitochondrial membranes, block the transport of nuclear-encoded mitochondrial proteins to mitochondria, interact with mitochondrial proteins, disrupt the electron transport chain, increase reactive oxygen species (ROS) production, cause mitochondrial damage, and prevent neurons from functioning normally. Recent scientific research has identified multiple mechanisms of amyloid-beta interaction with mitochondria at different mitochondrial compartments: the outer mitochondrial membrane, intermembrane space, inner mitochondrial membrane, and the matrix. It is well known that the involvement of amyloid-beta-induced mitochondrial dysfunction in AD pathogenesis, a vicious cycle as well as several vicious circles within the cycle, each accelerating the other, can be drawn emphasizing the Alzheimer mitochondrial cascade hypothesis.

The brain is vulnerable to oxidative stress owing to its high lipid content, its relatively high oxygen metabolism, and its low levels of antioxidant defenses [26]. One of the most interesting events in AD is that mitochondrial oxidative stress occurs early in AD progression, before the onset of amyloid-beta pathology [27, 28]. Oxidative stress was also reported in the mitochondria of other tissues different to the brain such as platelets and fibroblasts from AD patients [29, 30].

Free radicals (compounds with an unpaired electron) or ROS are a normal part of metabolism. Mitochondria are the major source of ROS, and, in fact, mitochondrial dysfunction as well as hypometabolism has long been implicated in the onset of the familial and sporadic forms of AD [31]. mtDNA defects have also been linked to an increased incidence of AD [32]. Quantitative morphometric, molecular, and cellular analysis of mitochondria shows increased abnormal and damaged mitochondria in AD [33, 34]. Energy deficiency and mitochondrial dysfunction have been recognized as a prominent, early event in AD.

Mitochondrial abnormalities have been found both in neurons and astrocytes [35–37], suggesting that both neurons and astrocytes might be damaged by free radicals in the AD brain. Superoxide radicals might be produced in mitochondrial electron transport chain complexes I and III [38] and in components of the Krebs cycle, including a-ketoglutarate dehydrogenase [39]. In addition, superoxide radicals might be generated in the outer mitochondrial membrane. H_2_O_2_ and superoxide radicals, released from the mitochondrial matrix and from the inner and outer mitochondrial membranes, might be carried to the cytoplasm and, ultimately, might lead to the oxidation of cytoplasmic proteins [26].

**Figure 2:**
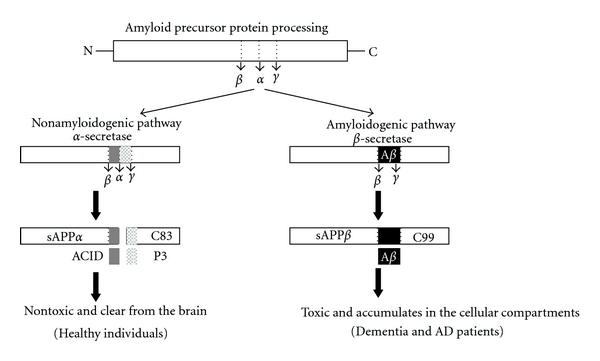
APP processing in nondemented healthy individuals and AD patients. APP processing occurs by two pathways: a beta-secretase-based amyloidogenic and alpha-secretase-based nonamyloidogenic pathway. In the nonamyloidogenic pathway (left), cleavage occurs by alpha-secretase within the amyloid-beta domain and generates the large soluble N-terminal fragment (sAPP*α*) and a non-amyloidogenic C-terminal fragment of 83 amino acid residues (C83). Further cleavage of this C-terminal fragment by *γ*-secretase generates the nonamyloidogenic peptide (P3) and APP intracellular domain (ACID). These products are nontoxic. The non-amyloidogenic *α*-secretase pathway occurs in over 90% of humans, and these individuals generally do not develop dementia. In the amyloidogenic pathway (right), cleavage occurs by **β*-*secretase at the beginning of the amyloid-beta domain and generates a soluble N-terminus fragment (sAPP**β**) and amyloidogenic C-terminal fragment of 99 residues (C99). This C-terminal fragment is further cleaved by *γ*-secretase and generates amyloid-beta. The amyloidogenic pathway occurs in approximately 10% of total humans, and these individuals might develop dementia and AD.

Several lines of evidence suggest that APP and amyloid-beta are factors contributing to mitochondrial dysfunction in AD (Figure 2). Mitochondria were found to be the target both for amyloid precursor protein (APP) that accumulates in the mitochondrial import channels and for amyloid-beta that interacts with several proteins inside mitochondria and leads to mitochondrial dysfunction [30]. Multiple lines of evidence support APP and amyloid-beta as contributing factors to mitochondrial dysfunction in AD: both APP and amyloid-beta are present in mitochondrial membrane and interact with mitochondrial proteins, block mitochondrial import channels, impair mitochondrial transport, disrupt the electron transfer chain, increase ROS levels, and cause mitochondrial damage.

With regard to localization of APP in mitochondria, it was demonstrated that APP formed stable 480 kDa complexes with the translocase of the outer mitochondrial membrane 40 (TOM40) import channel and a supercomplex of approximately 620 kDa with both mitochondrial TOM40 and the translocase of the inner mitochondrial membrane 23 (TIM23) import channel TIM23 in an N (in mitochondria) -C (out of cytoplasm) orientation [40]. Interestingly, in brain tissues of AD-affected subjects, APP localized with mitochondria fraction, associated to TOM40 and TIM23, in a translocation-arrested manner, that may prevent import of *de novo *synthesised nuclear-encoded mitochondrial protein, such as subunits of the electron transport chain [27].

In agreement with the intracellular localization of APP, cell studies showed mitochondrial accumulation of amyloid-beta in AD patients and APP mouse transgenic mouse brain [28]. In transgenic APP mice, mitochondrial amyloid-beta accumulation increased at around 4 months of age, well before the formation of plaques [41]. In total, these findings are further in line with the recently proposed hypothesis of an intracellular amyloid-beta toxicity cascade which suggests that the toxic amyloid-beta species intervening in molecular and biochemical abnormalities may be intracellular soluble aggregates instead of extracellular, insoluble plaques [42].

APP and amyloid-beta may block mitochondrial translocation of nuclear-encoded proteins [32], such as components of the electron transport chain [43–45], impairing mitochondrial function. Intramitochondrial amyloid-beta is able to perturb mitochondrial function in several ways by directly influencing extracellular transport chain complex activities [46], impairing mitochondrial dynamics [11], or disturbing calcium storage [47, 48], thus increasing apoptotic pathways [49]. Moreover, amyloid-beta interacts with mitochondrial matrix components inducing an improper mitochondrial complex function leads to a decreased mitochondrial membrane potential of the organelle [50] and impairing ATP formation [51].

In APP processing, monomeric amyloid-beta forms oligomers in synaptic terminals. Oligomeric amyloid-beta is hypothesized to enter in the mitochondria by penetrating the membrane because amyloid-beta is enriched at synaptic terminals [52]. In support of the hypothesis that APP and amyloid-beta enter mitochondria, several studies have found APP and its derivatives (monomeric and oligomeric forms of amyloid-beta) in mitochondrial membranes [53, 54]. Amyloid-beta normally interact with the mitochondrial matrix protein, amyloid-beta-binding alcohol dehydrogenase (ABAD), leading to mitochondrial dysfunction [9]. Caspersen et al. [41] showed amyloid-beta in mitochondria from postmortem brain specimens of AD patients and an accumulation of amyloid-beta in the brain mitochondria from APP mice. With digitonin fractionation analysis of isolated mitochondria from APP mice revealed amyloid-beta in outer and inner mitochondrial membranes and matrix and that mitochondrial amyloid-beta decreases cytochrome oxidase activity and increases free radical production and carbonyl proteins [28].

Further, in the amyloid interactions in mitochondrial proteins, two more studies found that APP interacts directly with mitochondrial proteins. It was demonstrated that mitochondrial ATP synthase subunit is a binding partner of the extracellular domain of APP and amyloid-beta [50]. Transfection of APP-deficient neuroblastoma cells with APP resulted in increased surface localization of the ATP synthase a-subunit and in extracellular APP and amyloid-beta inhibiting the extracellular generation of ATP. In another study, the authors found that nonglycosylated full-length and C-terminal-truncated APP accumulates in the protein import channels of mitochondria of human AD brains but not in age-matched controls [27]. The accumulation of APP across mitochondrial import channels inhibited the entry of nuclear-encoded cytochrome c-oxidase subunits IV and Vb proteins and was associated with decreased cytochrome oxidase and increased free radical production.

## 5. Mitochondrial DNA (mtDNA) Changes in AD

It is well documented that mtDNA changes are responsible for aging phenotypes [55–57]. For example, many tissues from aged individuals have a lower respiratory function compared with those from younger individuals. It has been hypothesized that ongoing oxidative damage to mtDNA may be the underlying mechanism for cellular senescence [58]. Since mtDNA repair mechanisms are limited and because mtDNA is situated in close proximity to the site of ROS production, mtDNA is more vulnerable to oxidative damage than nuclear DNA [59]. With age, oxidation of mtDNA increases compared to nuclear DNA leading to an age-dependent accumulation of mtDNA mutations [60].

Point mutations and deletions in mtDNA are highly prevalent in aged cells, and there is evidence that 8-hydroxy-2- deoxyguanosine (damaged DNA) is more prevalent in aged tissues [55]. Further, mice carrying an mtDNA mutation (in the DNA polymerase-g gene) showed features of aging and reduced lifespan, suggesting that mtDNA changes are crucial for aging phenotypes [56].

Defects in mtDNA have not only been found in elderly persons without AD but also in AD patients [61, 62] and have been associated with decreased cytochrome oxidase activity in non-AD aging and aging AD brains. One recent study found that somatic mtDNA control region mutations are elevated in AD patients [63]. These mutations would lead to an overall reduction in mtDNA copy number which would result in a decrease in oxidative phosphorylation. In addition, a mutation that affects L-strand transcription was also discovered. This mutation inhibits complex I respiration which leads to increased ROS production, decreased membrane potential, and subsequent calcium deregulation. The effects of these mutations may lead to opening of the mitochondrial permeability transition pore and subsequent neuronal death.

Increased ROS levels act at multiple levels to impair mitochondrial function: they induce mtDNA mutations [64] that consequently negatively influence mitochondrial function [65], enhance amyloid-beta production by guiding APP cleavage pathway toward the amyloidogenesis [66], increase lipid peroxidation [67, 68], activate mitophagy, leading to a reduced mitochondrial number [36], and augment tau hyperphosphorylation and NFT formation impairing organelle trafficking and neuronal function finally leading to apoptosis.

Using quantitative RT-PCR techniques, it was measured mRNA expression of 11 mitochondrial-encoded genes in patients with early AD and with definite AD, as well as in age-matched control subjects. This interesting analysis revealed a downregulation of mitochondrial genes in complex I of OXPHOS (oxidative phosphorylation) in brain of AD patients. Complex I showed a down regulation of mitochondrial genes, whereas complexes III and IV showed increased mRNA expressions in these AD brains, suggesting a great demand on energy production [35]. In a previous paragraph, we reported a decrease of cytochrome oxidase in the mitochondria from platelets, fibroblasts, and brains of AD patients. To compensate for the loss of cytochrome oxidase, mitochondrial-encoded genes might be activated in the surviving brain neurons of AD, for those patients this chain of events has been interpreted as a compensatory response [26].

## 6. Mitochondria and Synaptic Damage in AD

Synaptic degeneration is an early pathological feature in AD and is closely correlated to impaired cognitive function and memory loss. Recent studies suggest that involvement of amyloid-beta peptide in synaptic mitochondrial alteration underlies these synaptic lesions. Based on recent findings in human AD subjects, AD animal models, and AD cellular models, synaptic mitochondria undergo multiple malfunctions including amyloid-beta accumulation, increased oxidative stress, decreased respiration, and compromised calcium handling capacity, all of which occur earlier than changes seen in nonsynaptic mitochondria prior to predominant AD pathology. Of note, the impact of amyloid-beta on mitochondrial motility and dynamics exacerbates synaptic mitochondrial alterations.

Mitochondrial number is indeed very high in neurons, and mitochondria are especially enriched in synapses. Due to the limited glycolytic capacity of neurons, these cells are highly dependent on mitochondria function for energy production. Thus, the importance of synaptic mitochondria in supporting synapses and the high vulnerability of synaptic mitochondria to amyloid-beta make them a promising target of new therapeutic strategy for AD.

Synaptic mitochondria are synthesized in neuronal soma; they are then transported to dendrites and axones, are distributed abundantly around synapses where mitochondria modulate calcium balance, and actively provide energy to fuel the synaptic function [69, 70]. If mitochondria localized in the cell body are damaged or are otherwise degraded, such as by aging or by amyloid-beta, these defective mitochondria might be transported to synaptic terminals by natural mitochondrial trafficking, where they produce low levels of ATP owing to their degradation. Synaptic mitochondria thus undergo constant activation to maintain synaptic function. Defects in synaptic mitochondria obviously compromise synaptic function being very vulnerable to accumulative damages.

Mitochondrial dysfunction and synaptic damage are early pathological features of AD. Gillardon et al. found amyloid-beta oligomers in synaptosomal mitochondrial fractions and decreased energy metabolism in AD transgenic mice [71]. Abnormalities of mitochondrial function, including decreased mitochondrial respiration, ROS generation, and hypometabolism, occur in the AD brain [39, 72] and in brains of AD mouse models [73, 74]. Amyloid-beta accumulation in the synapses directly disturbs mitochondrial function, causing oxidative stress, decreased ATP, and increased Ca^2+^ influx [9, 75]. Furthermore, the interaction of mitochondrial amyloid-beta with its binding proteins, such as ABAD and CypD [9, 75], exacerbates amyloid-beta-induced mitochondria and neuronal stress and malfunction.

Further, it was recently showed that amyloid-beta impaired synaptic mitochondrial distribution, axonal mitochondrial mobility, and increased axonal mitochondrial fragmentation. Interestingly, anterograde movements were more susceptible to amyloid-beta insult. These promising findings are in agreement with those of recent studies indicating that amyloid-beta causes rapid and severe impairment of mitochondrial transport [47] and alters mitochondrial dynamics [76].

Synaptic terminals are sites of high energy demand and require high levels of cellular ATP for neurotransmitter exocytosis and the potentiation of neurotransmitter release. These studies suggest that in an amyloid-beta-rich environment, overt mitochondrial dysfunction occurs and that mitochondria provide a direct site for amyloid-beta-mediated cellular perturbation, causing synaptic mitochondrial dysfunction in AD. However, it is not yet known whether amyloid-beta accumulates predominantly in synaptic mitochondria and whether synaptic mitochondria enriched for amyloid-beta are more vulnerable, although it is well known that the increase in oxidative damage exhibited by synaptic mitochondria might affect neurotransmission and synaptic damage and loss and might be ultimately responsible for cognitive decline in AD patients.

In view of the critical role of synaptic mitochondria in energy production, synaptic calcium buffering, and regulation of synaptic function, impaired movement to and sequestration of mitochondria at synapses by amyloid-beta could be a mechanism of the pathogenesis at the distal synapses, together with early changes in synaptic mitochondrial function.

## 7. Mitochondrial Dysfunction in Choroid Plexus

In AD is very well documented the intracellular deposits of amyloid-beta in the brain parenchyma; however, amyloid-beta also accumulates in choroid plexus epithelial cells [77] and in cerebrovascular walls, where it induces blood-brain barrier disruption [78]. Several studies have shown that amyloid-beta alters transmembrane and cytoplasmic tight junction proteins in brain microvessel endothelial cells and choroid plexus, which ultimately leads to disruption in the integrity of the blood-brain barrier [79, 80].

Abnormal patterns of mitochondrial stress protein expression are found in the choroid plexus and BBB of AD subjects. There was a trend towards increased expression of HSP60, a mitochondrial stress protein, compatible with mitochondrial pathology recently documented in choroid plexus of AD [81]. It was recently described in choroid plexus homogenates from 27 cases with AD-related pathology in different stages (stages I/0, III-IV/0-B and V-VI/B-C) increased carboxyethyl-lysine (CEL) and carboxymethyl-lysine (CML) expression in AD cases stages IVB and V-VI/B-C when compared to controls and cases with AD-related pathology. Interestingly, the authors suggest that other factors in addition to local fibrillar amyloid-beta were associated with oxidative damage in the choroid plexus. Though two-dimensional gel electrophoresis in combination with mass spectrometry identified other proteins as targets of increased oxidative damage in AD (tyrosine 3/tryptophan 5-monooxygenase activation protein, zeta polypeptide, tropomyosin 3 isoform, and apolipoprotein A-II) [82].

Recent results from our laboratory have suggested direct relationship between amyloid-beta accumulation at the choroid plexus epithelium and the development of functional and structural dysfunctions [77, 83]. In addition, we demonstrated the existence of a link between amyloid-beta-induced choroid plexus cell death, increased production of nitric oxide (NO), and mitochondrial dysfunction in the choroid plexus of patients with AD and APP/PS1 mice [83]. In AD patients and APP/Ps mice, we found an alteration induced by amyloid-beta of the enzyme activity of the respiratory chain complex IV in the choroid plexus epithelial cells [84]. Accumulation of amyloid-beta peptides is a critical event in the pathology of AD, because they induce multiple neurotoxic effects, including mitochondrial dysfunction. Based on these results, we considered a reduction of amyloid-beta with gelsolin such as primary therapeutic target [85].

Gelsolin is an amyloid-beta binding protein that inhibits apoptosis, although the underlying mechanism is unclear. We observed that gelsolin reduces brain amyloid-beta burden in the APP/Ps1 mice, accompanied by an inhibition of nitric-oxide production and cell death, not only in the choroid plexus but also in the cerebral cortex. Gelsolin levels restored the impaired mitochondrial activity, resulting in the increase of cytochrome c-oxidase activity [84].

Additionally, these result demonstrated that gelsolin plays an important role in decreasing amyloid-beta-induced cytotoxicity by inhibiting nitric oxide production and apoptotic mitochondrial changes. All these promising findings make gelsolin an appealing tool for the prophylactic treatment of AD.

## 8. Conclusions

Based on findings from *in vitro* and *in vivo* studies, it has been proposed that amyloid-beta has a significant role in synaptic dysfunction and cognitive decline in AD patients. Among other facts, amyloid-beta accumulates at synaptic terminals and enters the mitochondria, especially in the synaptic mitochondria. Mitochondria have been implicated in the neurodegenerative diseases and the onset and progression of age-associated diseases since decades. Mitochondria are the major source of energy for the brain [86]. The accumulation of mtDNA changes might increase ROS production and reduce mitochondrial ATP in an age-dependent manner. Recent studies of neurons from postmortem AD brain specimens and from transgenic AD mouse brain specimens suggest that oxidative damage induces amyloid-beta production.

Studies of postmortem brains from AD patients and transgenic mouse models of AD suggest that oxidative damage, induced by amyloid-beta, is associated with mitochondria early in AD progression. Amyloid-beta present within mitochondria may provide a direct link between mitochondrial dysfunction in AD and pathogenic amyloid-beta. Amyloid-beta associated with mitochondria may be deposited at several locations, and yet nobody has known if amyloid-beta enters only in presynaptic mitochondria, postsynaptic mitochondria, or both. Interestingly, amyloid-beta is not present exclusively on the outer mitochondrial membrane and also might be present at that site to influence the interaction of multiple cytosolic proteins (including those of the bcl2 family) with mitochondria, as well as affecting the receptor binding of cargo targeted for import into the organelle. Amyloid-beta and APP are reported to localize to mitochondrial membranes, block the transport of nuclear-encoded mitochondrial proteins to mitochondria, interact with mitochondrial proteins, disrupt electron transport chain activities, increase ROS production, cause mitochondrial damage, and prevent neurons from functioning normally.

The mechanisms of amyloid-beta and APP transport into mitochondrial membranes are unclear. Amyloid-beta accumulates at synaptic terminals and impairs synaptic function and also enters synaptic mitochondria and causes damage. Mitochondrial damage is expected to be greater in synaptic mitochondria than in cell body mitochondria. The damaged, synaptic mitochondria might not satisfy the high energy demands required at synapses, which might lead to impaired neurotransmission and, ultimately, to cognitive failure. Based on these concepts, therapies targeting basic mitochondrial processes, such as energy metabolism or free radical generation, or specific interactions of disease-related proteins with mitochondria, hold great promise and future. In AD, tremendous progress has been made in developing therapeutic strategies to decrease amyloid-beta production and toxicity. However, further research is needed to develop molecules that target intact amyloid-beta in brain neurons affected by AD. Further research is also needed to test the efficacies of mitochondrially targeted antioxidants in AD mouse models. Future experiments that focus on the functional association of mitochondria with APP and amyloid-beta might be useful for identifying mitochondrial drug targets.
